# Factors Determining Effective Orthokeratology Treatment for Controlling Juvenile Myopia Progression

**Published:** 2017-09

**Authors:** Qinghui KONG, Jiang GUO, Jing ZHOU, Yanling ZHANG, Xiaoyan DOU

**Affiliations:** 1. Dept. of Ophthalmology, Shenzhen Second Hospital, Shenzhen, Guangdong, China; 2. Dept. of Teaching and Research, School of Medical Technology and Nursing, Shenzhen, Guangdong, China

**Keywords:** Orthokeratology, Myopia, Retrospective analysis, Logistic regression analysis

## Abstract

**Background:**

We aimed to identify factors influencing the therapeutic outcome of orthokeratology on controlling juvenile myopia progression, and the risk factors for complications.

**Methods:**

Myopic patients (n=724) in Shenzhen Second Hospital from Jan 2011 to Jan 2016 fitted with orthokeratology lenses and followed-up for 6–65 months were reviewed retrospectively. Univariate and multivariate logistic regression analyses were used to screen for the factors that can improve treatment outcome and prevent the development of complications.

**Results:**

Patients where the orthokeratology treatment was effective displayed a shorter myopia time, smaller diopter and corneal curvature, larger corneal endothelium density, high proportion of overnight wear and longer wearing times compared with patients whose treatments were ineffective. Additionally, wearing Ortho-k for 6 or 12 months yielded improved corrective effect and achieved higher comfort level. Logistic regression analyses showed that myopia time, diopter, corneal curvature e value, corneal endothelium density, time with Ortho-k and corrective effect after wearing Ortho-k for 6 or 12 months were all independent factors influencing the treatment effects. Results showed corneal curvature, anterior chamber depth and central corneal thickness were independent risk factors.

**Conclusion:**

This study systematically identified the factors leading to effective treatments, and those carrying a risk for complications, to provide guidance for the prescription and follow-up of orthokeratology in the treatment of juvenile myopia.

## Introduction

China’s juvenile myopia rate ranks second place in the world. It is generally accepted that myopia is one of the main diseases leading to blindness (1). Treatment options to slow down the rate of myopia progression include medication, spectacles, Ortho-k and laser operation.

Wearing Ortho-k can be a safe and effective way to slow down the progression of myopia (2–3). Ortho-k can be personally designed according to the patients’ corneal shape and diopter. It is made from rigid and high permeability material with allows oxygen to reach the cornea, they are optically stable and reversible (4). Therefore, Ortho-k lenses can be worn for prolonged periods.

This study focused on identifying the factors influencing the therapeutic effects of orthokeratology for controlling juvenile myopia progression, and the risk factors for complications.

## Materials and Methods

### Subjects

Medical records of 724 patients diagnosed with myopia in Shenzhen Second Hospital (Shenzhen, Guangdong, China) from Jan 2011 to Jan 2016 and treated with Ortho-k were reviewed retrospectively. The following inclusion criteria were adopted: 1. No congenital diseases of eyes such as strabismus. 2. SER (spherical equivalent refractive) ≤ −6.00 D, flat meridian corneal curvature ≤ 50.00 D, corneal astigmatism ≤ −2.00 D, BCVA ≥ 0.5, and intraocular pressure within normal range. 3. No spectacles or Ortho-k wearing or ocular medication history, and no Ortho-k treatment contraindications such as dry eye or conjunctivitis. 4. Good patient compliance and sufficient clinical data.

There were 4 exclusion criteria: 1. Undergoing medical or surgical treatment during the period wearing Ortho-k. 2. Myopia progression influencing factors (like poor working posture) were not being corrected. 3. Unadvised change in Ortho-k lens model and brand. 4. Failure to be present at follow up.

The follow-up time ranged from 6 to 65 months, in the end 666 cases (92%) cases conformed to the inclusion criteria for the research and 58 cases were excluded.

This study was approved by the Ethics Committee of Shenzhen Second Hospital. Signed written informed consents were obtained from the patients and/or guardians.

### Methods

The clinical data collected for each case included gender, age at the start of Ortho-k treatment, myopia time (period from confirmed diagnosis to the first day wearing Ortho-k), UCVA (Uncorrected Visual Acuity) and BCVA (Best Corrected Visual Acuity) using the Standard logarithmic acuity chart (GB11533-89, China), diopters (auto-refractor NIDEK ARK-700A, NIDEK, JAPAN), sphere (D), cylinder (D), corneal curvature (Orbscan II Z, BAUSCH &LOMB, USA), corneal thickness, axial length (slit-lamp Topcon SL-1E,Topcon, JAPAN), intraocular pressure (non-contact tonometer TX-10, Canon, Japan), anterior chamber depth, central corneal thickness (specular microscope SP-3000P, Topcon, Japan), corneal endothelium density, Ortho-k model, wearing modality (overnight, daytime and/or flexible wear), time with Ortho-k (from the first day wearing it to the end of follow-up time), corrective effect after 6 and 12 months (UCVA and BCVA should be improved at least 1 unit), and comfort level (results of survey ranging from 10 as the most comfortable to 1 as the least comfortable level).

Complications encountered included infections, inflammation, corneal light and paropsis. The Ortho-k lenses used were Boston XD, with 128 oxygen permeability, 10.0–11.0 mm in diameter, 0.24 mm of optical zone center thickness, and anti-geometry four-arc designed inner surface; the manufacturers were OPPLE Chinese Cognex, US E&E and Japan ORTHO-K. According to the adopted treatment standards, the treatment was only regarded as effective when both UCVA and BCVA values improved by at least 1 unit.

### Statistical analysis

The SPSS20.0 software (Chicago, IL, USA) was used for statistical analysis. Measurement data were expressed as mean ± standard deviation, the individual sample t test was adapted for group comparison. The paired sample *t* test was used for comparison within the groups. Enumeration data were expressed as NNT or percentage. The χ^2^ test was used for group comparisons. The logistic regression model was applied to multivariate analysis, inclusion criteria α ≤ 0.10, elimination criteria β ≥ 0.05 and step back screening *P <* 0.05 were regarded as meaningful in the statistical analysis.

## Results

### Univariate analysis of therapeutic effect

The group where the treatment was effective had a shorter myopia time, smaller diopter and corneal curvature but larger corneal endothelium density, high proportion of overnight wear and longer wearing time compared to the group of cases where the treatment was ineffective (Table 1). In addition, those wearing Ortho-k for 6 or 12 months experienced a better corrective effect and achieved a better comfort level.

**Table 1:** Univariate analyses of therapeutic effect

**Groups**	**Effective (n=566)**	**Ineffective (n=100)**	**t/χ^2^**	***P***
Male/Female	267/299	52/48	0.793	0.373
Age start wearing Ortho-k (yr)	12.4±3.5	13.2±3.6	0.245	0.865
Myopia time (months)	5.2±2.6	8.6±3.2	4.523	0.032
UCVA	0.6±0.2	0.6±0.3	0.063	0.925
BCVA	0.8±0.3	0.8±0.3	0.057	0.956
Flat meridian (D)	−3.5±1.2	−4.6±1.5	4.321	0.035
Sphere (D)	−3.8±1.4	−3.7±1.3	0.125	0.869
Cylinder (D)	−0.7±0.2	−0.6±0.2	0.133	0.842
Astigmatism degree (D)	−0.8±0.3	−0.9±0.3	0.084	0.899
Corneal curvature e value (D)	0.5±0.1	0.7±0.2	4.627	0.030
Corneal thickness (μm)	516.4±45.2	515.2 ±52.3	0.215	0.765
Axial length (mm)	24.6±3.2	24.8±3.3	0.163	0.859
Intraocular pressure (mmHg)	16.8±3.5	17.5±3.7	0.212	0.765
Anterior Chamber Depth (mm)	3.6±0.5	3.6±0.7	0.086	0.924
Central corneal thickness (mm)	536.2±42.6	534.7±35.9	0.326	0.695
Corneal endothelium density (number/mm^2^)	3564.8±562.4	3120.6±635.7	5.627	0.019
Ortho-k model [case (%)]			0.362	0.834
China	152	24		
US	216	40		
Japan	198	36		
Wearing modality [case (%)]			126.385	0.000
Overnight wear	475	32		
Daytime wear	35	24		
Flexible wearing	56	44		
Time with Ortho-k (months)	13.5±4.6	6.7±2.2	6.537	0.000
Corrective effect after 6 months	1.6±0.4	0.8±0.3	5.213	0.020
Corrective effect after 12 months	1.3±0.5	0.6±0.3	5.064	0.025
Comfort level (values)	7.5±1.3	5.3±1.2	5.326	0.017

### Multivariate analysis of therapeutic effect

According to the regression analysis, myopia time, diopter, corneal curvature e value, corneal endothelium density, time with Ortho-k and corrective effect after wearing 6 or 12 months are independent factors with effects on the treatment outcome (Table 2).

**Table 2:** Multivariate analysis of therapeutic effects

**Factor**	***β***	**Wald**	***P***	**OR**	**95% CI**
Myopia time (months)	0.065	3.215	0.021	1.236	0.064∼2.563
Diopter	0.123	3.562	0.018	1.421	0.752∼2.215
Corneal curvature e value (D)	0.127	4.632	0.007	1.968	1.134∼3.265
Corneal endothelium density (number/mm^2^)	−0.245	3.205	0.024	1.128	0.039∼3.524
Time with Ortho-k (months)	0.326	3.364	0.020	1.864	1.162∼2.549
Corrective effect after 6 months	−0.125	4.121	0.012	2.134	1.329∼3.258
Corrective effect after 12 months	−0.084	4.526	0.010	2.521	1.864∼3.219

### Univariate analysis of complications

During the study 53 cases experienced serious complications, 3 of them experienced eye infections, 32 suffered from conjunctival congestion, edema, allergy and corneal aseptic infiltration and 6 cases had paropsis.

In all of these 53 cases, the use of Ortho-k lenses was stopped and symptomatic treatment was applied. A few patients with mild reactions continued to wear Ortho-k after being treated symptomatically. The corneal curvature in the group of patients who experienced complications was larger, the anterior chamber depth smaller, the central corneal thickness thinner and the time wearing the lenses longer compared to the same parameters in those cases without complications (Table 3).

**Table 3:** Univariate analysis of complications

**Group**	**Complications (n=53)**	**Without Complication (n=613)**	**t/χ^2^**	***P***
Corneal curvature (D)	46.3±2.4	43.8±3.5	5.217	0.024
Anterior Chamber Depth (mm)	3.5±0.5	3.6±0.8	4.328	0.035
Central corneal thickness (mm)	531.7±35.4	538.2±56.7	5.845	0.009
Time with spectacle (months)	15.8±3.7	12.7±3.5	5.624	0.012

### Multivariate analysis of complications

The logistic regression analysis showed that corneal curvature, anterior chamber depth and central corneal thickness are all independent risk factors for complications (Table 4).

**Table 4:** Multivariate analysis of complications

**Factor**	***β***	**Wald**	***P***	**OR**	**95% CI**
Corneal curvature	−0.214	3.215	0.032	1.214	0.063∼2.527
Anterior Chamber Depth	0.325	3.426	0.027	1.326	0.076∼2.748
Central corneal thickness	0.142	3.658	0.024	1.457	0.095∼3.124

## Discussion

The mechanism behind orthokeratology’s therapeutic effects is because the lens base arc curvature of the lenses used is smaller than the corneal optical zone. The lens, therefore, compresses the central area of the cornea mechanically elongating the focal point and forcing the corneal tissue to move from central area to the surrounding area. At the same time, there is a certain gap between the lens and the central corneal area. Tear fluid in this gap attains the same function as the lens increasing the focus of the compressed central area of the cornea to correct refractive errors (5).

Myopia time, diopter, corneal curvature e value, corneal endothelium density, time with Ortho-k and corrective effect after wearing 6 or 12 months are all independent factors which can affect the treatment outcome. An earlier start age of myopia and shorter myopia time in a patient are factors determining a better corrective effect (6). However, a recent study found no relationship between the starting age of myopia and a corrective effect (6). This may be because a developing eye can have myopia progressing very rapidly (7). A diopter is an optical performance index to evaluate the imaging in the eyes. Because the Ortho-k changes the corneal shape by using mechanical compression, a reversible feat, the vision improvement is limited and not permanent (8).

Ortho-k can improve the UCVA and BCVA by an average of 2.5±0.8 and 1.1±0.6 lines, respectively (9–10). Generally, the best vision improvement can be achieved after wearing Ortho-k for 6–18 months. The smaller the diopter, the better the improvement in corrective effect. The high oxygen permeability material of the lenses has a significant effect on the corneal endothelium’s growth and metabolism. As the Ortho-k wearing time increases, the density of corneal endothelium decreases, and the effects on vision improve (11). Due to the necessity of prolonged periods wearing the Ortho-k, understanding by the patient and good adherence are of critical importance on improving the corrective effects (12). Under the supervision of parents and follow-up of attending physician, adherence to a proper schedule wearing the Ortho-k is the guarantee of vision improvement in children.

Corneal curvature, anterior Chamber Depth and central corneal thickness are all independent factors, which can influence the occurrence of complications (13). Ortho-k lenses make direct contact with the cornea, conjunctiva and tear film, they cannot only change the vision mechanics, but also have an impact on the local cell survival environment. Moreover, a larger corneal curvature, smaller anterior chamber depth, thinner central corneal and closer lens contact with eyes will result in an increasing possibility of regional hypoxia (13–14). The incidence of bacterial and viral infections in these cases will also increase to about 0.06 to 0.15% (15).

Therefore, Ortho-k should be strictly sterilized before being used to improve the vision and comfort level, and to prevent localized infections. Additionally, during the follow-up periods in our study, we found that a mild dry eye or an ocular surface inflammation (like vernal conjunctivitis) could also negatively influence the vision corrective outcome.

## Conclusion

This research systematically recorded and analyzed the many independent factors that influence the effects of orthokeratology for controlling juvenile myopia progression and complications. More importantly, this study offers a valuable reference for clinicians on choosing right candidates, predicting corrective effects and preventing the occurrence of complications when prescribing orthokeratology.

## Ethical considerations

Ethical issues (Including plagiarism, informed consent, misconduct, data fabrication and/or falsification, double publication and/or submission, redundancy, etc.) have been completely observed by the authors.
